# Nuclear organization and 3D chromatin architecture in cognition and neuropsychiatric disorders

**DOI:** 10.1186/s13041-016-0263-x

**Published:** 2016-09-05

**Authors:** Alejandro Medrano-Fernández, Angel Barco

**Affiliations:** Instituto de Neurociencias (Universidad Miguel Hernández-Consejo Superior de Investigaciones Científicas), Av. Santiago Ramón y Cajal s/n. Sant Joan d’Alacant, 03550 Alicante, Spain

**Keywords:** Nuclear structure, Chromatin, Epigenetics, Neuronal plasticity, Chromosomal interactions, Neuropsychiatric disorders

## Abstract

The current view of neuroplasticity depicts the changes in the strength and number of synaptic connections as the main physical substrate for behavioral adaptation to new experiences in a changing environment. Although transcriptional regulation is known to play a role in these synaptic changes, the specific contribution of activity-induced changes to both the structure of the nucleus and the organization of the genome remains insufficiently characterized. Increasing evidence indicates that plasticity-related genes may work in coordination and share architectural and transcriptional machinery within discrete genomic foci. Here we review the molecular and cellular mechanisms through which neuronal nuclei structurally adapt to stimuli and discuss how the perturbation of these mechanisms can trigger behavioral malfunction.

## Introduction

In the search for mechanisms that underlie behavioral plasticity, functional and structural changes at synapses are at the core of the theoretical framework. Processes such as long-term potentiation (LTP) or synaptogenesis are thought to be crucial for the adaptation of neuronal circuits to changing environmental conditions [1]. Both stimulus-driven transcriptional responses [2] and different forms of epigenetic regulation [3] are known to participate in these processes. However, only recently high-order chromatin architecture has been implicated in the neurobiology of behavior [4]. Cell biology studies have revealed that the compartmentalization of chromatin dictates the location of specific genes within the neuronal nucleus, thereby conditioning the mechanisms controlling their transcription [5]. The complexity and cellular heterogeneity of neuronal tissue make technically difficult the investigation of the contribution of activity-induced changes in chromatin architecture to neuronal plasticity. However, as technological advances enable deeper insight into the genomic landscape of neurons, increasing evidence indicates that individual genes do not work in isolation; instead, they share niches and machinery within the cell nucleus that sustain coordinated regulation. The levels of regulation include changes in nuclear geometry and subnuclear structures, dynamic interactions of structural proteins and the transcription machinery with chromatin, the relocation of genes into transcriptionally active or repressive areas, and chromatin loopings that activate regulatory sequences. In the following sections, we review recent studies that have begun to unveil the contribution of these novel mechanisms to neuronal plasticity, and highlight how their malfunction can contribute to the on-set or further development of neuropsychiatric disorders.

## Neuronal nuclear structure and its regulation by neuronal activity

In eukaryotic nuclei, DNA is wrapped around an octameric histone core comprising of two copies of each of the canonical histones H2A, H2B, H3 and H4. This basic structure, known as a nucleosome, is repeated along the double-stranded DNA, with a fifth type of histone (the linker histone H1) bridging together consecutive nucleosomes. In this fashion, long DNA strands condense with architectural proteins to form chromatin. Based on the level of compaction we can distinguish three main forms of chromatin. These forms differ biochemically with respect to the presence of specific post-translational modifications (PTMs) at the histone tails and to the binding of structural proteins. Euchromatin, a transcriptionally active form is characterized by permissive marks such as the trimethylation of histone 3 at lysine 4 (H3K4me3), and the acetylation of different lysine residues at the histone tails. In contrast, heterochromatin is a transcriptionally silent form, and is decorated by repressive epigenetic marks. It can be found in two different functional states: constitutive heterochromatin that is characterized by DNA methylation at CpGs and histone H3 trimethylation at lysine 9 (H3K9me3), and facultative heterochromatin, which, as suggested by its name, can harbor transcriptional activity and is marked by H3K27me3 [6].

**Fig. 1:**
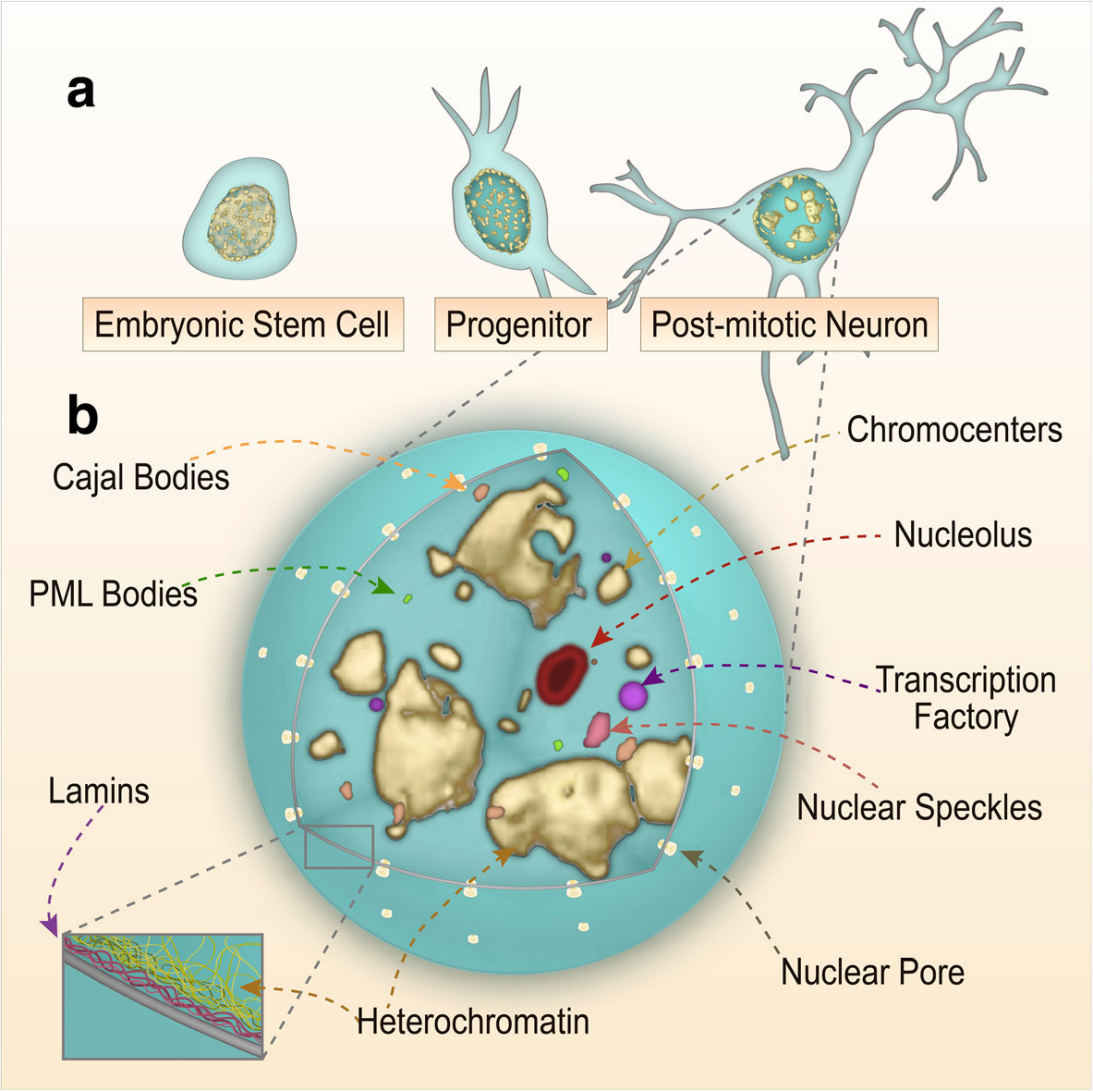
Nuclear structure and sub-compartments. **a.** Developmental changes as seen with DAPI staining (in yellow). The nucleus of an embryonic stem cell is euchromatic and relatively homogeneous. Heterochromatin foci (centromeres and telomeres) become more evident in neuronal progenitors. Mature neurons present fewer and denser chromocenters (adapted from microscopy images in [8]). **b.** Different types of nuclear bodies can be found in the nucleus of post-mitotic neurons

Although the folding of chromatin fibers during cell division is very similar among all cells [7], the spatial organization of the chromatin in the interphasic nucleus can greatly differ. Thus, during neuronal maturation, centromeric constitutive heterochromatin foci from different chromosomes are reduced in number, and cluster in larger foci known as chromocenters [8, 9] (Fig. 1a). These structures are depleted of the facultative heterochromatin marks H3K27me3 and H3K9me2, and the active isoforms of RNA Polymerase II (RNAPII), indicating that they lack the potential to be transcriptionally active [10]. In parallel to chromocenter formation, chromosome territories are distributed in the interior of the nucleus, defining regions with different gene densities in which gene-poor regions are generally located at the periphery while gene-rich regions are found in the interior of the cell nucleus [11]. Recent studies on the nuclear architecture of chicken neurons have revealed a more extreme form of radial nuclear organization in which chromocenters are radially aligned between the peripheral heterochromatin and DNA-depleted areas in the central nucleoplasm [10]. Notably, some highly specialized neurons, such as the retinal rods of nocturnal mammals, present an inverted distribution of the heterochromatin that could contribute to maximize light transmission trough photoreceptors thereby serving a unique function in nocturnal vision [12].

Apart from chromocenters and peripheral heterochromatin, the interphasic neuronal nucleus is structurally complex [13] (Fig. 1b). Based on conventional microscopy techniques, we can define three major components: the nuclear lamina and associated heterochromatin, the nucleoplasm that is defined by a fine and relatively homogeneous granular matrix, and the different internal macrostructures that disrupt this granular matrix. In the following sections we will discuss each of these components and their responses to neuronal activation.

### Nuclear envelope and lamina

Nuclear architecture and genome organization depend on the integrity of the nuclear envelope, a boundary that separates the cytoplasm from the nucleoplasmic reticulum. This boundary is composed of two phospholipid bilayers spanned at intervals by proteins that act as nuclear pores. The nuclear envelope is not an inert barrier, it participates in different processes including gene regulation and the transport of ions and macromolecular cargos [14]. Its geometry in neurons is rather plastic and responds to neuronal activity [15]. In the case of hippocampal neurons, there are both spherical and highly infolded nuclei featuring different degrees of complexity, with nuclear infoldings being antagonistically regulated by synaptic and extrasynaptic NMDA receptors [16]. Infolded nuclei typically have larger surfaces accompanied by an increase in nuclear pore complexes (NPC) that facilitates calcium influx and the transport between the nuclear and cytosolic plasmas.

Internally attached to the nuclear envelope is the nuclear lamina, whose main components are the lamin proteins A/C, B1 and B2 [17]. These proteins form a scaffold and bind to peripheral chromatin, playing an essential role in transcriptional regulation. Cellular biology studies have shown that the lamin composition of the nuclear envelope changes throughout neuronal differentiation. While primary progenitors have lamin A/C, B1 and B2 in equal amounts, neuroblasts have more B1 and some B2, and mature neurons preferentially express B2, some A/C, and little B1 [18]. Genetic experiments in mice have demonstrated that lamins B1 and B2, despite their great sequence homology, have unique roles in the developing brain, and that increased production of one does not compensate for the loss of the other [19, 20].

Lamin-associated chromatin domains (LADs) are enriched in transcriptional and epigenetic repressors [21]. Although the attachment of chromatin to the nuclear lamina has been found to promote transcriptional repression [17], this relationship is not strict. In fact, genes in both the margin and the center can be expressed, although peripheral genes are less likely to be transcribed than inactive genes dissociated from the lamina [22, 23]. Although little is still known about signal transduction across the nuclear envelope in neurons, a recent study on the role of the calcium signaling modulator Sigma-1 receptor (Sig-1R) demonstrated that the translocation of this receptor from the endoplasmic reticulum into the nuclear envelope upon cocaine administration may contribute to the addictive properties of this drug. Once in the nucleus, Sig-1R recruits chromatin-remodeling molecules such as lamin A/C, barrier-to-autointegration factor (BAF) and histone deacetylases (HDAC) to specific loci, shutting down the expression of monoamine oxidase B (MAOB), an enzyme that is dramatically upregulated during withdrawal and whose inhibition may contribute to the reinforcing properties of cocaine [24].

### Nuclear bodies

Nuclear bodies are subnuclear divisions that lack a membrane. In addition to the afore discussed chromocenters, which are the most prominent type of nuclear body in mature neurons, one can also typically find (i) a single nucleolus where rRNA transcription takes place, (ii) the Cajal bodies (CBs) that are adjacent to the nucleolus and are the site for small nuclear ribonucleic protein (snRNP) assembly, (iii) nuclear speckles that are highly enriched in splicing factors, and (iv) promyelocytic leukemia (PML) bodies that hold unknown functions [25] (Fig. 1b). As discussed for the nuclear lamina, these structures can undergo dramatic changes upon neuronal activation. For example, the amyloid precursor protein, intracellular domain–associated protein-1 (AIDA-1d) is a post-synaptically localized protein that translocates into the nucleus after synaptic stimulation. This translocation increases the number of nucleoli and may eventually promote protein synthesis [26]. Notably, nucleolar integrity has been shown to be necessary for LTP [27]. PML bodies are also sensitive to changes in activity; they tend to cluster into fewer, but denser and larger foci as a result of epileptic activity or exposure to behaviorally stressful conditions such as restraint [28]. In turn, the disruption of CBs and splicing speckles has been also associated with pathological states [29, 30], but the molecular machinery underlying these changes and its contribution to pathoetiology remains unknown.

### The nucleoplasm

The nucleoplasm is not an inert and homogeneous matrix filled with euchromatin fibers as once thought. Static electron microscopy images have since been challenged by the dynamic scenario revealed by molecular studies that explore short and long-range interactions between DNA sequences that are located thousands of bases apart or even in different chromosomes [31]. The use of super-resolution microscopy has recently allowed the direct visualization of fibers rich in nucleosomes, which can be frequently grouped into “clutches” and are interspaced with nucleosome-depleted DNA. The density of these clutches differs across cell types with stem cells having a lower density compared to mature neurons [32].

Fine submegabase 3D interactions are essential for neuronal commitment and are also likely to contribute to the regulation of gene expression during neuronal plasticity processes. We will discuss in the next section the novel techniques available and the seminal studies investigating how neuronal activation causes changes in the fine structure of the nucleoplasm.

## Neuronal 3D genome organization and its regulation by neuronal activity

Given their small scale, transcription-related and activity-driven dynamic changes in chromatin fibers may escape structural analyses when employing microscopy techniques, but can be tackled by molecular studies investigating long and short-range chromosomal interactions [33]. This is the case for chromosome conformation capture (CCC or 3C) techniques that are used to analyze the organization of chromosomes in intact cells. Since the invention of this PCR-based technology in 2002 [34], the emergence of various next-generation sequencing (NGS)-based techniques has dramatically transformed our understating of genome architecture. For example, Hi-C enables CCC studies to be performed on a genomic scale, Chromatin Interaction Analysis by Paired-End Tag Sequencing (ChIA-PET) allows the determination of *de novo* long-range chromatin interactions genome-wide, and DNase I hypersensitive sites sequencing (DNase-seq), Formaldehyde-Assisted Isolation of Regulatory Elements (FAIRE)-seq and Assay for Transposase-Accessible Chromatin (ATAC)-seq allow the assessment of changes in DNA accessibility [33]. These novel NGS techniques in parallel with the aforementioned progress in cell imaging now provide us with an exceptional opportunity to interrogate neuronal chromatin dynamics [33, 35]. For example, FAIRE-seq has revealed major genomic reorganizations during both differentiation and neuronal stimulation [36], and ulterior Hi-C experiments have shown that topologically-associated domains (TADs) are organized into hierarchical domain-within-domain structures named metaTADs. Some of these metaTADs are remodeled during neuronal maturation while others remain unchanged, thereby supporting stability and at the same time that enabling the adaptability of specific loci [37].

### Loci relocation

A key level of genome organization is the movement of genes within the interior of the nucleus. Fundamental contributions in the eighties demonstrated chromosomal movements in seizure foci of the human cortex. These movements were found to affect particularly the X chromosome although they were independent of the patient’s sex [38]. Consistently, the induction of LTP in the hippocampus has been shown to provoke the clustering of satellite DNA in hippocampal neurons [39]. More recent experiments have further elaborated on the details of such chromosomal movements. For instance, in the case of *Bdnf*, it has been observed that upon kainate-induced seizures there is both a weakening of its interaction with the lamina as well as the relocation of one allele from the nuclear margin to deeper areas within the nucleus [40]. This relocation resulted in the colocalization of *Bdnf* alleles with poised RNAPII. Intriguingly, the detachment from the lamina persisted beyond the transient increase in transcription, which leaves open the possibility that this structural change could contribute to sensitization of affected neurons for ulterior reactivation [40]. A similar internalization of the *Bdnf* locus has also been reported to occur in neuronal cultures after depolarization [41]. These movements in the nucleoplasm correlate with the wave of active transcription that follows strong synaptic activation [41]. These gene movements resemble those reported to occur during neuronal differentiation. For example, when neural precursors acquire neuronal commitment, *ASCL1* (encoding for the Mash1 protein), along with other proneural genes, move from the nuclear periphery where they remain transcriptionally silent to the central nucleoplasm where they become transcribed [23, 42].

### Architectural proteins involved in chromatin loops, and long and short-range interactions

Enhancers are defined as regulatory sequences rich in transcription factor (TF) binding sites that regulate gene activation and are distal to the transcription start site (TSS) [43]. They are often located over 10 Kb from their respective genes, with 22 % of them being found more than 100 Kb away, and are usually identified by their enrichment in H3K4me1 and H3K27ac [22].

The expression of cell type-specific and brain region-specific genes often relies on enhancer sequences that act specifically only in those cells, while being methylated and inactive elsewhere [44]. Interestingly, these sequences are usually linked to a single promoter [31, 45] and often participate in intricate chromatin loops [46]. Indeed, promoter-enhancer architecture is essential in triggering activity-regulated transcriptional programs. In neurons, about 13,000 enhancers have been identified within a few Kb from TSSs [47]. Luciferase reporter assays have demonstrated productive elongation in these sequences, and led to the identification of enhancer RNAs (eRNA), a special kind of non-coding RNA (ncRNA) whose transcription is initiated near the center of the enhancer sequence [47]. Intriguingly, protein-coding genes associated with eRNAs are highly transcribed, and knocking down the eRNA dampens transcription of the neighboring genes [45]. Indeed, eRNA transcription is a proxy of 3D promoter-enhancer interactions because the release of nascent protein-coding RNA from the promoter needs Negative Elongation Factor (NELF) to bind eRNA and enter into productive elongation [48]. Recent genomic screens aimed to characterize enhancers that mediate activity-dependent transcription in mouse cortical neurons have underscored the importance of the TF Fos, which is itself subjected to regulation by neuronal activation, in the regulation of activity-driven gene programs [49]. In fact, the broad inducibility of *Fos* in the nervous system seem to rely in the action of at least five enhancers that surround the locus and differentially respond to various stimuli (e.g., membrane depolarization, BDNF binding and adenyl cyclase stimulation) [50].

Another type of regulatory sequence that relies on chromatin looping are the insulators. Insulators are described as chromatin regions that protect against the activating influence of distal enhancers associated with other genes [51]. The proteins CTCF (*aka* CCCTC-binding factor), mediator and cohesin are important components of the insulator complex that appear in distinct combinations depending on the range of interaction. CTCF and cohesin locate together in active regulatory sequences where they mediate long-range constitutive interactions. They are fundamental building blocks behind insulated chromosomal neighborhoods containing super-enhancers necessary for cell identity [52]. For instance, the presence of CTCF/cohesin marks megabase-sized TADs whose boundaries are usually constant among all cell types, although there can be cell-type specific subTAD organization [53]. Whereas cohesin is involved in regulation of tissue-specific transcription [54], CTCF plays a prominent role enabling chromatin looping through the pairing of sequences that contain its binding site [53, 55]. In turn, mediator and cohesin are found in short-range complexes that bridge enhancers and promoters. While mediator is necessary for the loading of enhancers with TFs and the formation of the initiation complex at the promoter [53], cohesin together with the “loader” protein Nipped-B-like protein (NIPBL) and other factors, brings DNA sequences together forming a ring structure that physically promotes their approximation [56]. The involvement of these proteins in neurodevelopment and cognition is supported by the finding that mutations in the encoding genes cause intellectual disability and severe neurodevelopmental defects (see below). Moreover, experiments in mice indicate that CTCF loss throughout developmental stages has been shown to cause neuronal death and deregulate neuronal differentiation [57], while ablation in postmitotic neurons caused growth retardation, abnormal hind-paw clasping, defects in somatosensory cortical maps, and reduced dendritic arborization and spine density [58].

### Poised RNAPII and transcription factories

The term transcription factory refers to discrete foci in the eukaryotic nucleus where transcription occurs [59]. These mega-structures promote physical interactions between genes that share the same regulatory machinery, which may enable their synchronous expression [60]. Consistent with this notion, genomic analyses indicate that TF binding can occur in nucleosome-depleted stretches of DNA lacking their canonical binding motifs through the interaction with other TFs and cofactors. Enhancer elements are also thought to form part of these mega transcription factor complexes [61] that are enriched in cohesin binding and strongly labeled with RNAPII antibodies [62]. It has been described in different immortalized human cell lines that loci highly enriched in RNAPII are often associated with looped chromatin in promoter-promoter interactions (the most common) or in the interactions between promoters and distal regulatory elements [61]. Single-gene complexes show a high intron/exon ratio, include looping conformations between promoters and enhancers, and usually are developmentally regulated and/or tissue-specific. Multigene complexes display interactions among several promoters and often also include enhancers. The genes found in multigene complexes are shorter (i.e., with lower intron/exon ratio), more enriched in GC, and are located in highly transcribed, gene-dense euchromatin regions that are rich in short interspersed nuclear elements (SINEs). Recent genomic studies indicate that, on average, there are more than eight genes per multigene complex [61], suggesting that promoter-promoter aggregates are a major feature of eukaryotic gene regulation. Such complexes provide the topological basis for common transcriptional regulation of gene groups. For instance, the 58 HIST1H genes located on chromosome 6 are organized into three complexes that further interact to form a larger complex [61]. It is tempting to speculate that poised plasticity-related genes share common transcription factories enriched in the same transcriptional regulators. This could occur through promoter-promoter interactions, which could ultimately synchronize their rapid expression due to higher-order chromatin structures in which RNAPII acts as a primary hub.

**Fig. 2:**
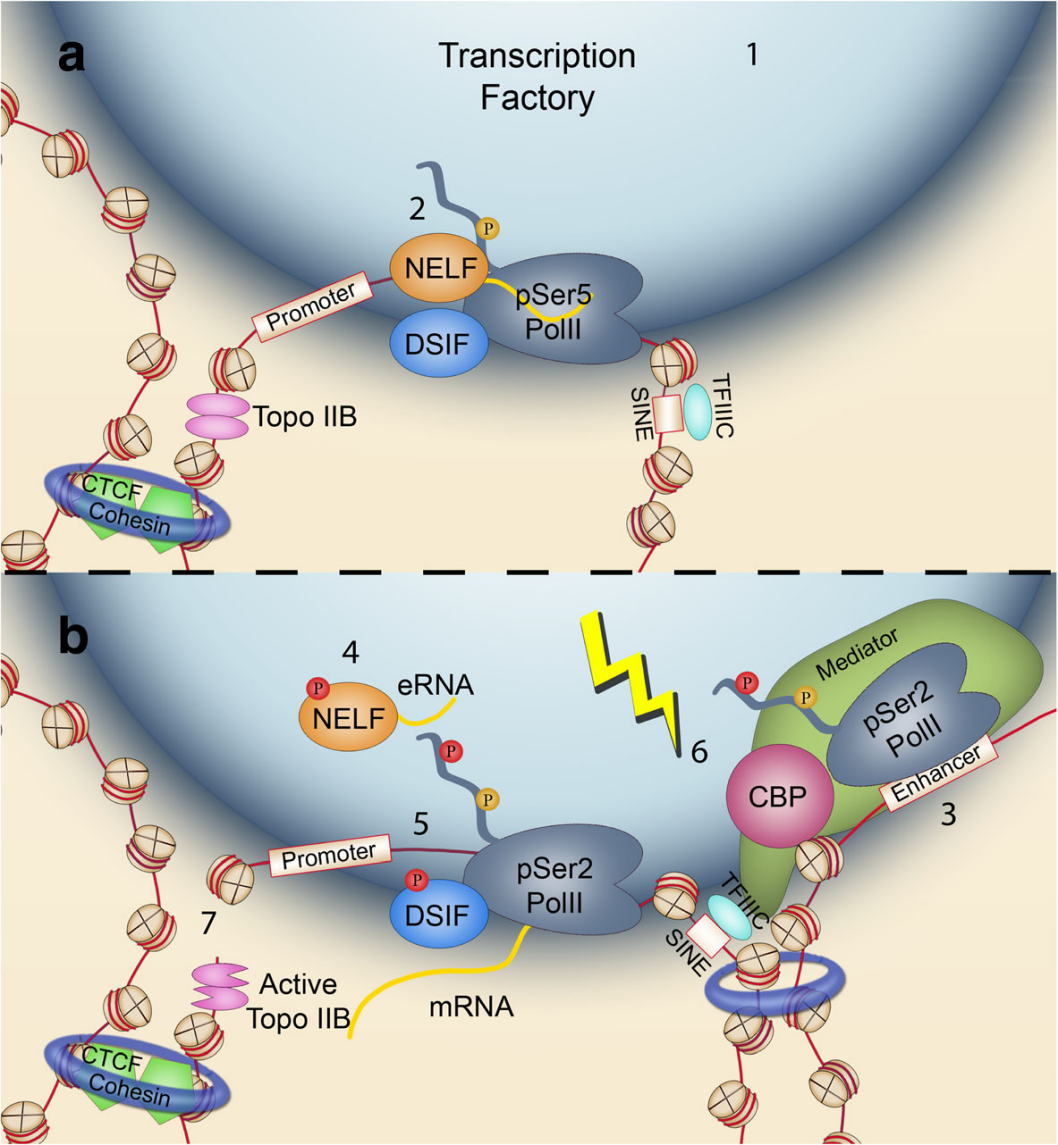
Activity-driven promoter/enhancer interactions leading to transcriptional elongation. **a.** In the basal state, RNAPII appears in transcriptional factories (an incompletely described proteinaceous body that is depicted in the scheme as a large blue globe) (1). The C-terminus of RPB1 has 52 tandem repeats of the heptapeptide YSPTSPS that contains two Ser residues that are dynamically phosphorylated. S5 phosphorylation (in orange) and the presence of the transcriptional repressors NELF and DSIF impede transcriptional elongation and stall RNAPII at gene promoters (2). **b.** Upon neuronal activity, distal enhancer sequences interact with the promoter thanks to the action of cohesin (3), which together with acetylated TFIIIC-bound SINEs mediates the relocation of plasticity genes. Enhancer acetylation requires the action of lysine acetyltransferases (4), such as CBP and p300, subsequently promoting their relocation. Transcriptional machinery (elongating RNAPII, the Mediator complex and TFs) binds to the enhancer element in order to transcribe eRNAs (5) that in turn bind to NELF and release it from the promoter. Finally, the phosphorylations of RNAPII (at Ser2), NELF and DSIF (red circles) would trigger productive elongation (6). In addition, it has been recently proposed that Topo IIB-mediated DSBs (upstream of the TSS) eliminate the loop that separates the promoter from the transcription factory (7)

The activity of these transcription factories is dynamically regulated by the phosphorylation of specific serine (Ser) residues at the C-terminus domain (CTD) of RPB1, the largest subunit of the RNAPII complex [63]. Although the phosphorylation of the Ser5 is required for transcription initiation, RNAPII remains incapable of elongation because NELF binding pauses nascent RNA synthesis and stalls RNAPII downstream of the TSS. To unlock stalling and engage in productive elongation, it is necessary the phosphorylations of RPB1 at Ser2 and the pausing factors NELF and DRB sensitivity–inducing factor (DSIF). Both repressors, upon phosphorylation, turn into positive regulators [64]. Recent Chip-seq experiments have revealed over 8000 gene promoters on which the RNAPII is stalled [65]. This state is often referred to as ¨poised polymerase¨ and has been shown to be a common feature of the TSSs of immediate early genes (IEGs) in neurons, enabling their rapid transcriptional recruitment upon neuronal activity [65] (Fig. 2a). A mechanism reported to contribute to the attachment of IEGs to transcription factories is the *de novo* acetylation of SINEs located around their promoter (Fig. 2b). This process is controlled by TFIIIC, a general TF that represses IEG transcription in the basal state. As such, the depletion of the TFIIIC subunit Gtf3c5 enhances the localization of IEGs in transcription factories, and subsequently favors their transcription and promotes dendritogenesis [41]. How TFIIIC mediates this effect is yet unclear, although it has been hypothesized that the acetylation of SINEs could be mediated through either its TFIIIC90 subunit that has intrinsic lysine acetyltransferase (KAT) activity [66], or by recruiting coactivators such as p300 that have KAT activity [41]. Another regulatory mechanism of activity-driven transcription may rely on the appearance of DNA double-strand breaks (DSBs). Thus, it has been recently shown that DSBs and the phosphorylation of histone variant H2AX occur at specific genomic loci, including the TSSs of several IEGs, after neuronal stimulation (Fig. 2b). Two hours later, DSBs were repaired and transcription was back to basal levels [67]. Intriguingly, although the artificial induction of DSBs mostly caused gene downregulation, some IEGs exhibited the opposite response suggesting a physiological role for DSBs in productive elongation.

## Chromatin architecture in neuropsychiatric disease

As introduced in previous sections, the neurons in some developmental and degenerative disorders often display gross nuclear aberrations, while psychiatric disorders have been associated with more subtle changes (Table 1). We discuss below some additional examples that demonstrate the strong connection between aberrant chromatin architecture in neurons and neuropathology.

**Table 1:** Neuropsychiatric conditions associated with disrupted nuclear organization and 3D chromatin architecture

Condition	Disruption	Reference
Alzheimer’s disease	Lamin B invaginations	[80]
Behavioral stress	PML clustering	[28]
Cocaine addiction	Sig-1R-mediated *MaoB* repression	[24]
Epilepsy	Chromosomal movements	[38]
Fragile X–associated tremor/ataxia syndrome	Heterochromatin condensation	[76]
Huntington’s disease	Super-enhancer dysfunction	[82]
Neurodegeneration	Disrupted CBS and speckles	[29, 30]
Seizures	PML clustering *Bdnf* relocation	[28, 40]
Alpha thalassemia/mental retardation syndrome X	*ATRX* mutation	[72]
Bipolar disorder	*PCDH*α enhancer SNP	[88]
Cornelia de Lange syndrome	*NIPRL*, *SMC1* and *SMC3* mutations	[70]
Fryns-Lujan syndrome	*MED12* mutation	[69]
ID, microcephaly and growth retardation	*CTCF* mutation	[71]
Impulsive-disinhibited personality	*SIRPB1* intronic deletion	[89]
Opitz-Kaveggia syndrome	*MED12* mutation	[69]
Post-traumatic stress disorder/depression	*FK506* intronic SNP	[93, 94]
Restless Legs syndrome	*MEIS1* enhancer SNP	[79]
Rett syndrome	*MECP2* mutation	[73]
Schizophrenia	*GRIN2B* enhancer SNP Microsatellite repeats in *NRG1* intron 1 *GAD1* enhancer-promoter dysfunction	[84, 85, 87]

This list is not exhaustive; it only presents those conditions discussed in the text. The rows under “Seizures” refer to conditions caused by mutations in architectural proteins or regulatory elements

### Neurodevelopmental disorders

Mutations in genes encoding proteins important for nuclear architecture (e.g. CTCF, cohesin and many epigenetic factors) frequently result in neurodevelopmental disorders [68]. This is the case of Opitz-Kaveggia syndrome and Fryns-Lujan syndrome which are both caused by mutations in *MED12* [69] that encodes a subunit of the mediator complex. Moreover, mutations in the genes encoding either NIPBL or the cohesin subunits SMC1 and SMC3 cause Cornelia de Lange syndrome [70], whereas mutations in the CTCF gene have been associated with intellectual disability (ID), microcephaly and growth retardation [71]. Further supporting the link between aberrant chromatin structure and ID, various genes encoding proteins that interact with heterochromatin, such as ATRX and MeCP2, are also linked to ID. Thus, mutations in the gene that encodes ATRX cause Alpha-Thalassemia X-Linked ID syndrome [72], while the loss of MeCP2 results in Rett syndrome [73] that manifests itself with ID and autistic traits. Neurons lacking MeCP2 show an abnormal number and size of nucleoli and chromocenters [74], and an aberrant distribution of pericentric heterochromatinization [75]. Other syndromes are also characterized by nuclear defects even though their etiology is not directly linked to nuclear organizers. For instance, hippocampal neurons with CGG repeat expansions in the *FMR1* gene, which give rise to fragile X-associated tremor/ataxia syndrome (FXTAS), accumulate more heterochromatin but in smaller foci [76].

Another type of genetic disorders associated with abnormal nuclear architecture are laminopathies in which the nuclear lamina is prominently disrupted. This group of disorders includes Hutchinson–Gilford progeria syndrome (HGPS) that is caused by mutations in the gene encoding lamin A [77]. Intriguingly, hippocampal nuclei of mouse models for this condition show abnormal lobulations and deep infoldings of the nuclear envelope, but gene expression and behavioral assays revealed no gross impairment [78], which indicates that neuronal nuclei can adapt to major perturbations in its structure. In contrast, as we will discuss in further detail for psychiatric conditions, other studies have shown that even local chromatin looping perturbations might lead to neurological symptoms. For example, the single nucleotide polymorphism (SNP) rs12469063 associated with Restless Legs syndrome, a sensorimotor neurological disorder, has been shown to cause looping perturbations and motor restlessness/hyperactivity in mouse models for this condition [79].

### Neurodegenerative disorders

Large-scale chromatin reorganization is often observed in neurons undergoing degeneration. Thus, irregularities in nuclear shape, particularly mediated by B-type lamins, have been described to precede heterochromatin relaxation, DNA damage and neurodegeneration in both *Drosophila* models of tauopathy and human samples from Alzheimer’s patients [80]. Furthermore, dispersion of the nuclear lamina is known to precede neuronal death and is a common feature seen in mouse models of Alzheimer’s disease [81]. Other alterations may not cause prominent structural changes but still affect function. For example, mouse models for Huntington’s disease (HD) exhibit diminished super-enhancer function of striatum-specific genes governed by Gata2 and display reduced H3K27ac and paused RNAPII binding [82].

### Psychiatric disorders

Aberrant chromatin loopings have been recently implicated in psychiatric disorders. For example, Akbarian and colleagues first found that overexpression of the histone methyltransferase Setdb1 caused the heterochromatinization of the promoter of *Grin2b* (encoding for a subunit of the NMDA receptor) and the loss of a loop tethering the promoter to a Setdb1 target site positioned 30 kb downstream of the TSS [83]. Further investigation of the same locus revealed that the SNP rs117578877, located at the distal arm of another *GRIN2B* loop, is often found in schizophrenic patients and correlates with impaired working memory and schizotypic features. Notably, isogenic deletions of loop-bound sequences in mice impaired cognitive performance and decreased *Grin2b* expression [84]. The same team has also reported abnormal chromosomal interactions at a second locus linked to schizophrenia. The formation of a chromatin loop between the TSS of *GAD1* (encoding an enzyme critical for GABA synthesis) and an enhancer sequence 50 Kb upstream was found reduced in the prefrontal cortex of schizophrenic patients [85]. A similar loop, sensitive to neuronal activation, was also detected in GABAergic neurons of mice. As a third example, it was recently demonstrated that a polymorphism affecting the interaction between the TSS of *FKBP5*, which encodes the co-chaperone FK506 binding protein 5, and enhancer sequences located in introns 2 and 7 is associated with an increased risk of developing stress-related psychiatric disorders after childhood trauma [86]. Another recent study has shown that microsatellite repeats in intron 1 of the gene encoding neuregulin 1 (*NRG1*), a putative schizophrenia susceptibility gene regulating the excitatory-inhibitory balance, are associated with an increase in NRG1 transcripts in the prefrontal cortex, suggesting that this region could function as a transcriptional enhancer. Intriguingly, the presence of these repeats correlated with an earlier age of onset of the symptoms. However, long-range interactions between the intronic sequence and the promoter remain to be experimentally proven [87]. There are additional examples suggesting that abnormalities in chromatin looping may be associated with conditions such a bipolar disorder [88] and impulsive-disinhibited personality [89], but molecular studies are still needed to prove the involvement of aberrant chromatin interactions in the etiology of these disorders.

### Cause or consequence

**Fig. 3:**
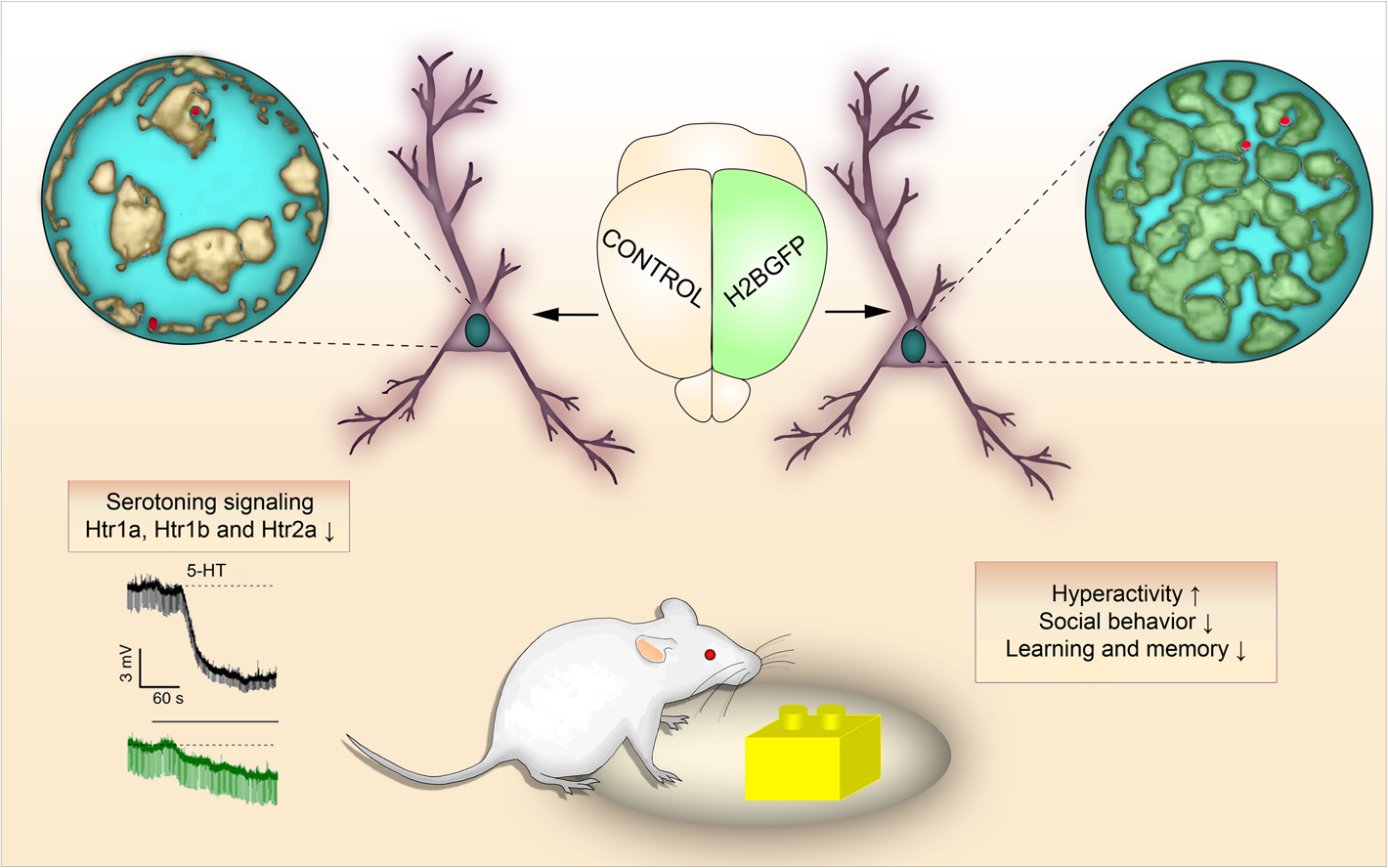
Chromatin perturbations cause behavioral impairments. The expression of the chimeric histone H2B-GFP causes dramatic changes in chromatin architecture, including the loss of peripheral heterochromatin, chromocenter declustering and changes in the texture of the nucleoplasm. This is likely due to stearic impediment of highly-packed tertiary chromatin fiber folding in heterochomatin by the protruding GFP tags. Remarkably, *Htr1a* alleles (red circles) relocated into the aberrant DNA foci, possibly explaining their downregulation and concomitant alterations in serotonin signaling and behavior

Given the difficulty of examining the specific contribution of chromatin conformation changes through gain- and loss-of-function experiments, most of the evidence discussed above is correlative. A recent study by our team investigating transgenic mice that express high levels of GFP-tagged H2B in forebrain principal neurons has provided evidence for a causal role of aberrant chromatin organization in the emergence of neuropsychiatric traits [90]. Neuronal nuclei in these mice presented an aberrant subnuclear pattern resulting from chromocenter declustering, a loss of perinuclear heterochromatin, heterodense nucleoplasm, and abnormal distribution of heterochromatic and euchromatic epigenetic markers (Fig. 3). The mice also exhibited a number of phenotypes related to neuropsychiatric symptoms, such as hyperlocomotor activity, impaired social interactions, nociception, sensorimotor gating and memory, and the downregulation of several serotonin receptor genes that sit in the edge of “gene desert” zones [90]. Suggestively, this topographical feature is conserved in the human genome and might relate to the susceptibility of these loci to epigenetic deregulation. In addition to this work, the aforementioned studies conducted by the Akbarian’s lab on chromosomal loops at schizophrenia-linked genes further support a causal link between the loss of specific chromatin loops, transcriptional deregulation and neuronal alterations [83–85].

Excitingly, the use of engineered transcription factors has recently demonstrated that the local manipulation of epigenetic profiles at a given gene is sufficient to control drug- and stress-evoked transcriptional and behavioral responses, thereby providing seminal evidence for a causative role for those epigenetic marks [91]. Similarly, CRISPR/Cas9 technology now enables direct manipulation of genome topology, opening up the possibility to conduct loss- and gain-of-function experiments exploring the role of altered DNA conformations in pathology and transcription [84]. For example, CRISPR/Cas9 has recently been used to change the orientation of two interacting chromosomal regions, demonstrating that the functionality *in vivo* of some enhancers carrying CTCF-binding sites relies on their relative orientation and the precise architecture of chromatin domains [92].

## Conclusions and prospects

As reviewed here, numerous studies have illustrated that nuclear architecture and genome topology are key for understanding neuronal function and dysfunction. Changes in subnuclear structures and chromatin loopings have been found to occur in different neuronal plasticity paradigms. Similarly, the disruption of chromatin structures is a landmark for numerous neurological disorders. Although such a disruption likely contributes to the onset of a disorder, a clear distinction between cause and consequence is still missing, except for some monogenic disorders (often associated with ID) caused by mutations in architectural proteins or regulatory sequences. Although the specific contribution of architectural proteins and the changes in 3D chromatin organization to neuroplasticity and neuropathology largely remain to be determined, new light will soon be shed now that novel techniques such as super-resolution microscopy, NGS-based techniques for the analysis of DNA conformation and CRISPR/Cas9-based epi-editing have emerged. These innovative approaches will facilitate a high resolution determination of the 3D organization of the genome, in parallel to a systems-level interrogation of the consequences of gene expression, the identification of loci associated with aberrant function, and even the manipulation of DNA conformations to promote or correct transcriptional changes.
